# Elevated third trimester corticosteroid levels are associated with fewer offspring infections

**DOI:** 10.1038/s41598-023-36535-0

**Published:** 2023-06-28

**Authors:** Nicole Prince, Rachel S. Kelly, Su H. Chu, Priyadarshini Kachroo, Yulu Chen, Kevin M. Mendez, Sofina Begum, Hans Bisgaard, Klaus Bønnelykke, Min Kim, Ofer Levy, Augusto A. Litonjua, Craig E. Wheelock, Scott T. Weiss, Bo L. Chawes, Jessica A. Lasky-Su

**Affiliations:** 1 Channing Division of Network Medicine, Department of Medicine, Brigham and Women’s Hospital and Harvard Medical School, 181 Longwood Avenue, Boston, MA 02115 USA; 2 Copenhagen Prospective Studies on Asthma in Childhood (COPSAC), Herlev and Gentofte Hospital, University of Copenhagen, 2820 Gentofte, Denmark; 3 Precision Vaccines Program, Boston Children’s Hospital and Harvard Medical School, Boston, MA USA; 4 Division of Pediatric Pulmonary Medicine, Department of Pediatrics, Golisano Children’s Hospital and University of Rochester Medical Center, Rochester, NY USA; 5 Unit of Integrative Metabolomics, Institute of Environmental Medicine, Karolinska Institutet, Stockholm, Sweden; 6 Department of Respiratory Medicine and Allergy, Karolinska University Hospital, Stockholm, Sweden; 7 Gunma University Initiative for Advanced Research (GIAR), Gunma University, Maebashi, Japan

**Keywords:** Metabolomics, Steroid hormones, Epidemiology

## Abstract

Respiratory infections are a leading cause of morbidity and mortality in early life, and recurrent infections increase the risk of developing chronic diseases. The maternal environment during pregnancy can impact offspring health, but the factors leading to increased infection proneness have not been well characterized during this period. Steroids have been implicated in respiratory health outcomes and may similarly influence infection susceptibility. Our objective was to describe relationships between maternal steroid levels and offspring infection proneness. Using adjusted Poisson regression models, we evaluated associations between sixteen androgenic and corticosteroid metabolites during pregnancy and offspring respiratory infection incidence across two pre-birth cohorts (N = 774 in VDAART and N = 729 in COPSAC). Steroid metabolites were measured in plasma samples from pregnant mothers across all trimesters of pregnancy by ultrahigh-performance-liquid-chromatography/mass-spectrometry. We conducted further inquiry into associations of steroids with related respiratory outcomes: asthma and lung function spirometry. Higher plasma corticosteroid levels in the third trimester of pregnancy were associated with lower incidence of offspring respiratory infections (P = 4.45 × 10^–7^ to 0.002) and improved lung function metrics (P = 0.020–0.036). Elevated maternal androgens were generally associated with increased offspring respiratory infections and worse lung function, with some associations demonstrating nominal significance at P < 0.05, but these trends were inconsistent across individual androgens. Increased maternal plasma corticosteroid levels in the late second and third trimesters were associated with lower infections and better lung function in offspring, which may represent a potential avenue for intervention through corticosteroid supplementation in late pregnancy to reduce offspring respiratory infection susceptibility in early life.

Clinical Trial Registry information: VDAART and COPSAC were originally conducted as clinical trials; VDAART: ClinicalTrials.gov identifier NCT00920621; COPSAC: ClinicalTrials.gov identifier NCT00798226.

## Introduction

Respiratory infections during childhood are a major cause of morbidity and mortality in early life^1^. In 2019 alone, there were an estimated 33 million episodes of acute lower respiratory infections in children between ages 0–5 years globally, resulting in > 100,000 preventable deaths^2^. Further, recurrent infections in early childhood can affect long-term health and increase the risk of developing chronic diseases later in life^3^. Recurrent respiratory infection incidence has been observed in subsets of highly susceptible children^4^, but this phenomenon is not well understood. Some epidemiological risk factors have been observed, such as daycare attendance, Caesarean section delivery, and maternal smoking in pregnancy^5^, but the underlying biology associated with increased infection proneness remains elusive. Exploring biological factors related to infection proneness could inform novel clinical strategies to prevent infection proneness.

The maternal environment during pregnancy can impact offspring respiratory^6,7^ and immune health^8^, and may similarly influence susceptibility to respiratory infections. Steroid signaling has been specifically implicated in respiratory and developmental outcomes that are closely related to early life respiratory infections^9,10^. However, studies addressing the connections between endogenous steroid levels in pregnancy and offspring respiratory and immune outcomes have produced conflicting results. While prenatal androgens promote fetal lung branching morphogenesis^11^, elevated levels may impair offspring immune response to pathogens post-birth^12^, Corticosteroids during pregnancy similarly represent a double-edged sword, as they are supplemented in late pregnancy to facilitate lung maturation^13^ but increase the risk of offspring respiratory diseases, such as asthma^9,14^. Given the intimate links between steroid levels in pregnancy and offspring respiratory and immune outcomes^15,16^, steroid metabolites may be relevant in childhood respiratory infection proneness and could represent targets with therapeutic potential.

In this exploratory study, we investigated associations between maternal plasma levels of androgen and corticosteroid metabolites and the number of respiratory infections that occurred in offspring. We incorporated two pre-birth cohorts of mother–child pairs from the Vitamin D Antenatal Asthma Reduction Trial (VDAART) and the Copenhagen Prospective Studies on Asthma in Childhood (COPSAC) to span early (10–18 gestational weeks [GW]), middle (22–26 GW), and late (32–38 GW) pregnancy periods. We conducted further inquiry into associations of steroid metabolites with asthma and lung function outcomes to assess potential broader implications of maternal steroids on offspring respiratory health. To our knowledge, ours is the first study to systematically assess the relationships between endogenous androgen and corticosteroid profiles during pregnancy and childhood infection proneness.

## Methods

### Cohort descriptions

VDAART was a randomized, double-blind, parallel-design study conducted at three study sites across the United States that preferentially recruited asthmatic mothers (ClinicalTrials.gov identifier: NCT00920621)^17^. VDAART recruited pregnant women at 10–18 GW and randomized them to Vitamin D supplementation at 4000 IU/day or placebo; all women received 400 IU/day vitamin D supplementation as part of usual pregnancy care. Blood samples were collected in EDTA tubes during enrollment between 10 and 18 GW and again between 32 and 38 GW; plasma was separated through centrifugation at 2000 RPM at 4 °C, and samples were stored at −80 °C until metabolomic profiling. For simplicity, throughout the manuscript, the earlier VDAART pregnancy time point (10–18 GW) will be referred to as “T1”, and the later VDAART pregnancy time point (32–38 GW) will be referred to as “T3”.

COPSAC was a single-center, pre-birth cohort study, as described previously^18^. As part of the COPSAC study, mothers were randomized to vitamin D_3_ supplementation of 2400 IU/day vs. placebo; all women received 400 IU/day of vitamin D_3_ as part of usual pregnancy care. Blood samples were collected in EDTA tubes from mothers during the 22–26 GW enrollment visit; plasma was separated and stored at -80 °C until processing^19^. Since this pregnancy time point occurs between the two VDAART time points, the COPSAC pregnancy period of 22–26 GW will be referred to as “T2” for simplicity.

### Infection proneness, asthma, and lung function outcomes in offspring

Respiratory infections in VDAART offspring were recorded between the birth of the child and age 6 years using standardized quarterly questionnaires completed by caregivers. In order to match the COPSAC reporting period, infections occurring only between birth and age 3 years were evaluated in this study. Respiratory infections in COPSAC offspring were recorded during scheduled clinical visits every 6 months to 1 year through parent report, beginning at birth until age 3 years. For both cohorts, the infection proneness outcome is a continuous variable representing the count of total reported respiratory infections reported, including upper respiratory infections, lower respiratory infections, colds, and ear infections. Asthma status in VDAART and COPSAC was recorded as positive if the child was diagnosed by a physician with asthma and/or recurrent wheeze any time between birth and age 6 years. Lung function spirometry measurements were collected in both cohorts at age 6 years, including forced expiratory volume in the first second (FEV_1_), forced vital capacity (FVC) and the ratio of FEV_1_/FVC; further details of spirometry measurement are available in Supplemental Methods S1.

### Steroid metabolite measurement by UPLC-MS/MS

Metabolomic profiles for VDAART and COPSAC plasma samples were generated by Metabolon, Inc. (NC, USA) using ultrahigh-performance liquid chromatography coupled to tandem mass spectrometry (UPLC–MS/MS). A total of 16 steroid metabolites were included in analysis, including 14 androgenic and 2 corticosteroid metabolites. Of these, 15 were Tier 1 assignments by Metabolon, with full structure identification matched to a standard. One androgenic steroid metabolite met putative Tier 2 identification by matching to a literature database, and is denoted with a superscript hash sign in Tables. Details of UPLC-MS/MS methods are available in Supplemental Methods S1.

### Metabolite associations with childhood outcomes

**Figure 1:**
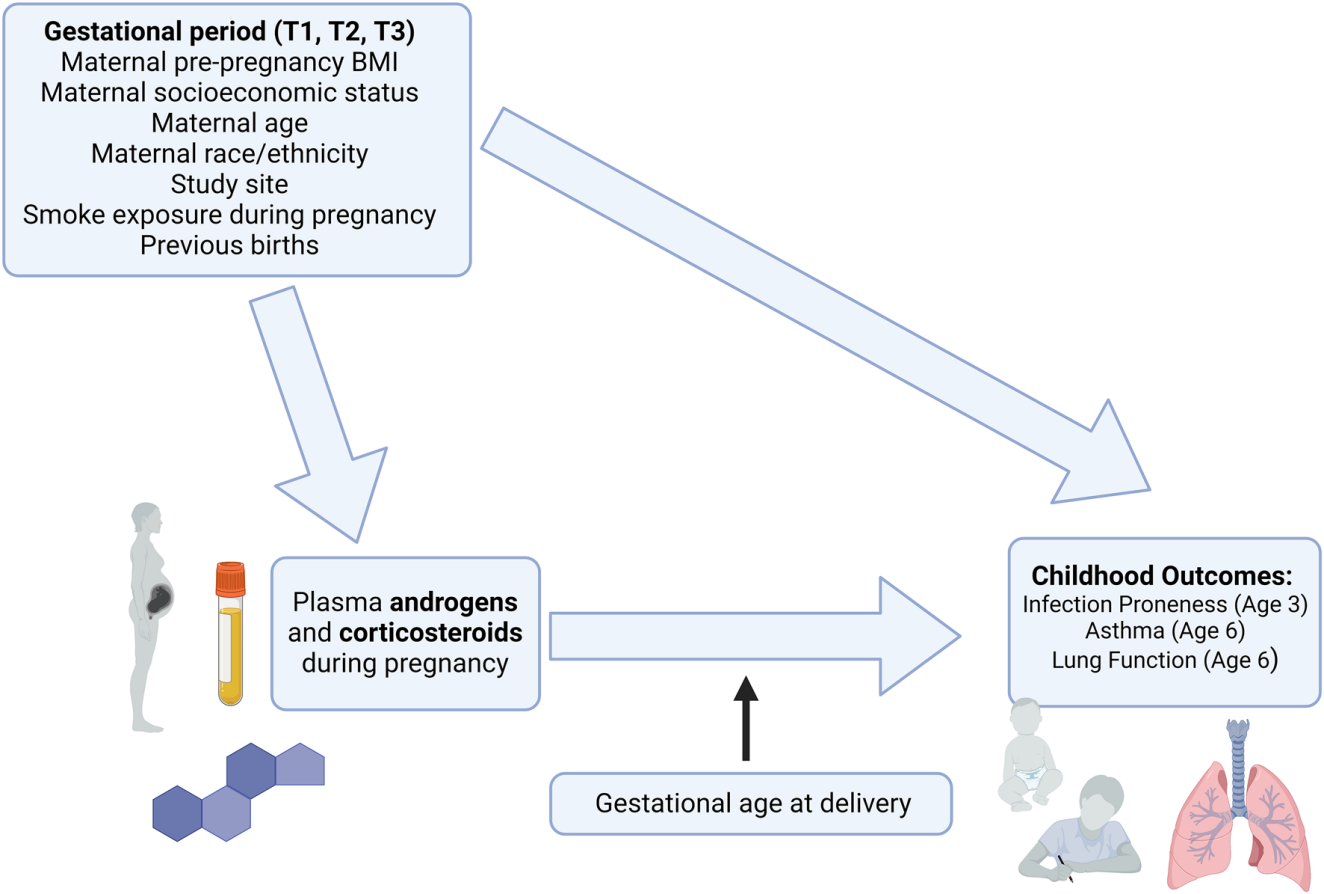
Overview of study design. Relationships between maternal steroids during pregnancy and childhood respiratory outcomes were estimated using two pre-birth cohorts of mother–child pairs. Regression models were adjusted for potential confounding variables, and mediation by gestational age at delivery was evaluated for steroid-outcome associations. Figure created in BioRender.

Regression analyses estimated associations between steroid metabolites and childhood outcomes at each of the three maternal time points using Poisson (infection proneness), logistic (asthma), or linear (lung function) regression models. Potential confounding factors were selected based on previous research (Fig. 1)^6^. VDAART models were adjusted for maternal age, race, ethnicity, pre-pregnancy BMI, vitamin D level (nanograms of 25 hydroxyvitamin D [25(OH)D] per milliliter of blood), maternal education status, birth order of child, and smoking during pregnancy; an additional variable for gestational days at collection was included for T1 only, as this time point spanned a longer eight-week period. COPSAC was homogenous with respect to race and ethnicity, so models used the same covariates as VDAART models except race and ethnicity. A restricted analysis of non-asthmatics mothers was also conducted for the infection proneness outcome to control for potential confounding by asthma status or asthma medication use (details in Supplemental Methods S1). Analyses were conducted using the stats package in R version 4.0.3^20^, and incidence rate ratios (IRRs), odds ratios (ORs), linear regression coefficients, and P-values are reported, as appropriate. Correction for false discovery rate (FDR) was performed using the Benjamini–Hochberg Procedure.

Mediation of the relationships between maternal steroids and infection proneness was assessed for the following potential mediators: gestational age at delivery, pregnancy-induced hypertension, gestational diabetes, pre-term labor, delivery mode, maternal asthma, and child daycare attendance during the first 3 years of life. Mediation analyses were performed using the stats^20^ and mediation^21^ packages in R 4.0.3.

### Ethics approval and consent to participate

All portions of this study were conducted in accordance with the Declaration of Helsinki. Local institutional review boards approved the protocol, and written informed consent was obtained from all individuals. The institutional review boards at each participating VDAART Clinical Center and the Data Coordinating Center at Brigham and Women’s Hospital approved protocols of the trial. This project was additionally approved by Brigham and Women’s Hospital IRB protocol 2018P000478. The COPSAC study was conducted in accordance with the Declaration of Helsinki and was approved by the Copenhagen Ethics Committee (KF 01-289/96) and the Danish Data Protection Agency (2008-41-1754).

## Results

A total of 774 mother–child pairs were included at VDAART T1 and 779 mother–child pairs were included at VDAART T3, which differed slightly in makeup due availability of blood samples for metabolomic profiling; 742 mothers had plasma samples at both time points. VDAART T1 and T3 were similar in all respects other than gestational stage and vitamin D level at time of blood collection (P < 0.001; Table 1). COPSAC mothers had lower BMI (P < 0.001) and were older (P < 0.001) than VDAART mothers; COPSAC offspring were delivered later than VDAART offspring (P < 0.001), and vitamin D levels in COPSAC mothers were significantly different from either VDAART time point (P < 0.001). COPSAC mothers demonstrated higher socioeconomic indices of income (P < 0.001) and education level (P < 0.001). There were also fewer asthmatic mothers in the COPSAC cohort (26.6%) than in VDAART (40.6% and 39.9% at T1 and T3, respectively). Children in VDAART experienced an average of 5.2 respiratory infections per year during follow-up, and COPSAC children experienced an average of 5.0 respiratory infections per year during follow-up. A total of 349 (45.1%) of children in VDAART experienced asthma/wheeze by age 6 years while 149 (21.8%) of COPSAC children were diagnosed with asthma by a doctor by age 6 years.

**Table 1:** VDAART and COPSAC cohort characteristics by gestational period.

Cohort characteristics	VDAART		COPSAC
	T1: 10–18 GW (n = 774)	T3: 32–38 GW (n = 779)	T2: 22–26 GW (n = 729)
Maternal variables			
Pre-pregnancy BMI kg/m^2^, mean (SD)	28.4 (7.7)	28.3 (7.7)	24.0 (5.9)
Age at enrollment yrs, mean (SD)	27.3 (5.5)	27.4 (5.5)	31.9 (4.5)
Gestational days at blood collection, mean (SD)	99.4 (19.2)	237.9 (11.6)	170.2 (6.2)
Race, n (%)			*NA*
Black	336 (43.4)	340 (43.6)	–
White	315 (40.7)	314 (40.3)	–
Other	123 (15.9)	125 (16.0)	–
Ethnicity, n (%)			*NA*
Hispanic or Latino	213 (27.5)	214 (27.5)	–
Not Hispanic or Latino	561 (72.5)	565 (72.5)	–
Study site, n (%)			*NA*
Boston	234 (30.2)	226 (29.0)	–
San Diego	259 (33.5)	269 (34.5)	–
St. Louis	281 (36.4)	284 (36.5)	–
Income category, n (%)^1^			
Low	331 (56.2)	329 (55.9)	65 (9.5)
Medium	171 (29.0)	172 (29.2)	364 (53.0)
High	87 (14.8)	88 (14.9)	258 (37.6)
Education category, n (%)^2^			
Low	474 (61.2)	482 (61.9)	54 (7.8)
Medium	191 (24.7)	183 (23.5)	444 (64.4)
High	109 (14.1)	114 (14.6)	191 (27.7)
Vitamin D level at time of collection (ng/mL), mean (SD)	22.9 (10.3)	33.0 (14.6)	30.1 (10.2)
Mothers in 4000 IU/day treatment group, n (%)	387 (50.0)	391 (50.2)	293 (51.0)
Mothers in 2.4 g/day fish oil group, n (%)	–	–	340 (49.7)
Maternal Asthma Diagnosis, n (%)	314 (40.6)	311 (39.9)	192 (26.6)
Smoking during pregnancy, n (%)^3^	117 (15.1)	120 (15.4)	58 (8.3)
C-section delivery, n (%)	228 (29.5)	233 (29.3)	161 (21.9)
Child variables			
Gestational age at delivery weeks, mean (SD)	39.0 (2.0)	39.2 (1.6)	39.8 (1.9)
Number of older siblings, mean (SD)	0.9 (1.1)	0.9 (1.1)	0.9 (1.0)
Daycare attendance, n (%)	360 (46.5)	353 (45.3)	676 (93.0)
Number of respiratory infections, 0–3 years mean (SD)	15.6 (8.8)	15.6 (8.9)	14.9 (9.3)
Respiratory infections/year, mean (SD)	5.2 (2.9)	5.2 (3.0)	5.0 (3.1)
Child asthma diagnosis, n (%)	349 (45.1%)	344 (44.6%)	149 (21.8%)

*Yrs* years, *SD* standard deviation, *BMI* body mass index.

^1^Income in VDAART was categorized based on reported household yearly income in USD: Low (< $50,000/year), Medium ($50,000-$100,000/year), or High (> $100,000/year); Income in COPSAC was categorized based on reported household yearly income in Euros: Low (< €50,000/year), Medium (€50,000-€110,000/year), or High (> €110,000/year).

^2^Maternal educational status was categorized based on maximum education level reported and categorized in VDAART as: Low (primary school, secondary school, or some college/junior college), Medium (technical/trade school or bachelor’s degree), or High (graduate degree); in COPSAC, education levels were categorized as: Low (primary school, secondary school, or college graduate), Medium (trade school or bachelor’s degree), or High (Master’s or other graduate degree).

^3^In VDAART, smoking or exposure to smoking in the home was recorded during enrollment questionnaires; in COPSAC, maternal smoking during pregnancy was recorded by COPSAC physicians.

### Maternal steroid associations with infection proneness

Associations between maternal corticosteroids and offspring infection proneness differed across pregnancy time periods. At VDAART T1, neither corticosteroid was associated with infection proneness at P < 0.05 (Table 2). At COPSAC T2, elevated cortisone was associated with reduced offspring infections (P = 0.007), and at VDAART T3, elevated levels of both cortisone and cortisol were associated with reduced offspring infections (P-values: 4.45 × 10^–7^ and 0.002, respectively); these associations retained significance at FDR < 0.05. In the restricted analysis of non-asthmatic mothers, only cortisone at VDAART T3 was associated with lower numbers of infections at P < 0.05 (Supplemental Table S1). However, increased cortisol showed a borderline association with reduced infections in the restricted analysis at T2 (P = 0.071). The relationships between third trimester corticosteroids and offspring respiratory infections were partially mediated by offspring gestational age at delivery (GA). At VDAART T3, 23.3% of the total effect of cortisol on infection proneness was mediated by GA (P = 0.002), and 9.6% of the total effect of cortisone on infection proneness was mediated by GA (P = 0.002). Pregnancy-induced hypertension, gestational diabetes, pre-term labor, delivery mode, maternal asthma, and child daycare attendance during the first 3 years of life, but none of these variables significantly mediated these relationships at P < 0.05 (data not shown).

**Table 2:** Associations between maternal steroid metabolites and offspring infections.

Steroid metabolite	VDAART T1 (N = 774) 10–18 GW			COPSAC T2 (N = 729) 22–26 GW			VDAART T3 (N = 779) 32–38 GW		
	IRR (95% CI)	P-value	FDR	IRR (95% CI)	P-value	FDR	IRR (95% CI)	P-value	FDR
16a-hydroxy DHEA 3-sulfate	1.01 (0.97,1.05)	0.561	0.641	0.97 (0.92,1.01)	0.157	0.236	1.04 (0.99,1.08)	0.091	0.290
Androstenediol (3beta,17beta) monosulfate (1)	1.01 (0.97,1.06)	0.530	0.641	*1.04 (1,1.08)*	*0.081*	*0.136*	1.01 (0.98,1.05)	0.570	0.684
Androstenediol (3beta,17beta) monosulfate (2)	1.02 (0.99,1.06)	0.219	0.396	**1.05 (1.02,1.08)**	**0.002**	**0.007**	0.98 (0.95,1.01)	0.181	0.364
Androstenediol (3alpha, 17alpha) monosulfate (3)	**1.04 (1.01,1.08)**	**0.021**	**0.171**	*1.04 (1,1.08)*	*0.064*	*0.136*	**1.05 (1.01,1.08)**	**0.016**	**0.062**
Androstenediol (3alpha, 17alpha) monosulfate (2)	**1.05 (1.01,1.09)**	**0.005**	**0.086**	1.01 (0.98,1.04)	0.361	0.451	**1.06 (1.02,1.09)**	**0.002**	**0.013**
Androstenediol (3beta,17beta) disulfate (2)	**1.05 (1,1.09)**	**0.040**	**0.212**	*1.05 (1,1.11)*	*0.076*	*0.136*	1.03 (0.98,1.07)	0.250	0.364
Androstenediol (3beta,17beta) disulfate (1)	1.03 (0.98,1.07)	0.223	0.396	**1.07 (1.03,1.12)**	**0.001**	**0.005**	1.02 (0.98,1.06)	0.373	0.498
5alpha-androstan-3beta,17beta-diol disulfate	1 (0.96,1.04)	0.995	0.995	**1.08 (1.04,1.13)**	**1.21 × 10**^**–4**^	**0.001**	1.02 (0.99,1.06)	0.210	0.364
5alpha-androstan-3beta,17alpha-diol disulfate	1.02 (1,1.05)	0.053	0.213	1 (0.98,1.03)	0.814	0.854	1 (0.97,1.03)	0.971	0.971
5alpha-androstan-3alpha,17alpha-diol monosulfate	1.02 (0.99,1.05)	0.193	0.396	**0.9 (0.86,0.94)**	**1.06 × 10**^**–6**^	**1.59 × 10**^**–5**^	1.01 (0.98,1.04)	0.606	0.684
Epiandrosterone sulfate	1.01 (0.98,1.05)	0.461	0.614	**1.06 (1.02,1.1)**	**0.004**	**0.011**	1.02 (0.99,1.06)	0.249	0.364
Andro steroid monosulfate C19H28O6S (1)*	1.01 (0.98,1.04)	0.601	0.641	1 (0.95,1.04)	0.854	0.854	0.99 (0.95,1.03)	0.641	0.684
Dehydroepiandrosterone sulfate (DHEA-S)	1.03 (0.99,1.07)	0.111	0.301	1.01 (0.97,1.06)	0.557	0.643	1.03 (0.99,1.06)	0.200	0.364
Androsterone sulfate	1.02 (0.98,1.06)	0.294	0.471	NA	NA	NA	1.03 (0.99,1.06)	0.128	0.342
Cortisone	1.05 (0.99,1.11)	0.113	0.301	**0.9 (0.85,0.97)**	**0.002**	**0.007**	**0.9 (0.86,0.94)**	**4.45 × 10**^**–7**^	**7.11 × 10**^**–6**^
Cortisol	1.02 (0.97,1.09)	0.414	0.602	0.97 (0.91,1.03)	0.254	0.346	**0.93 (0.89,0.97)**	**0.002**	**0.013**

Incidence rate ratios (IRRs), P-values, and FDR-corrected P-values of associations are shown at T1, T2, and T3 time points. Associations at P < 0.05 are bolded. Associations at 0.05 < P < 0.1 are italicized for COPSAC.

Elevated maternal androgens at T1 and T2 were generally associated with higher numbers of offspring infections, but results were inconsistent across individual androgen metabolites (Table 2, Supplemental Table S1). Across time points, 3 of 14 androgenic steroid metabolites at T1 (P-value range: 0.005–0.040), 5 of 13 at T2 (P-value range: 1.06 × 10^–6^ to 0.004), and 2 of 14 at T3 (P-value range: 0.002–0.016) were associated with increased infection proneness at P < 0.05. No androgen metabolites were consistently associated across all 3 time periods at a nominal P < 0.05 threshold. Trends in analyses restricted to non-asthmatic mothers were largely consistent in the direction of effect (Supplemental Table S1). Relationships between androgenic steroids and infections were not mediated by gestational age at delivery.

### Maternal steroids associations with other outcomes

Associations of steroids with childhood asthma were largely non-significant (Supplemental Table S2) except at VDAART T1. Increased levels of corticosteroids at T1 were associated with higher risk of offspring asthma (cortisone: OR [95% CI] 1.77 [1.10, 2.84], P = 0.019; cortisol: OR [95% CI] 1.77 [1.08, 2.9], P = 0.024), but these results were not consistent with infection proneness findings. No other associations were observed with asthma at a nominal level of P < 0.05.

Cortisone and cortisol at T3 were associated with improved %FEV_1_ (P = 0.036 and 0.020, respectively), but these associations did not survive correction for multiple testing. The associations between androgens and lung function were inconsistent across individual metabolites and gestational periods, but results are available in Supplemental Tables S3, S4, and S5.

## Discussion

Our study represents a novel investigation of associations between endogenous maternal steroid levels during pregnancy and respiratory infection proneness in offspring, which have not previously been explored. Maternal steroid levels can significantly impact fetal respiratory and immune health^10^, but the relationships of steroids with early life infections are not as well understood. Existing literature has established connections between maternal steroid levels during pregnancy with asthma^22^ and immune^8^ outcomes separately, but respiratory infection proneness represents an overlap in these respiratory and immune domains that remains elusive. Moreover, prior studies have generated conflicting findings regarding the beneficial and harmful effects of these steroids^9,11–14^, leaving their impact on childhood respiratory infections unclear. Our results suggest that higher corticosteroid levels in late pregnancy were beneficial in reduced offspring respiratory infections. This study represents an initial step towards understanding the contributing factors that influence childhood infection proneness, which may hold clinical relevance in efforts towards reducing the burden of early life respiratory infections.

Increased levels of corticosteroids in the third trimester of pregnancy were robustly associated with reduced offspring respiratory infections in this study. Corticosteroids have been well described for their clinical utility in respiratory health^23^, and our results support a role for corticosteroids in alleviating the burden of early life respiratory infections. Although some associations between corticosteroids and offspring infections did not meet nominal significance in the restricted analysis of non-asthmatic mothers, this may be a consequence of reduced statistical power, as the directions of effect were consistent with overall analyses. Steroid supplementation in pregnancy promotes fetal lung maturation, particularly in pregnancies at high risk of preterm labor^13^, and our formal mediation findings indicate gestational age at delivery was a significant mediator of the relationship between third trimester corticosteroids and infections, which further emphasizes the role of corticosteroids close to term. As corticosteroids are utilized in high risk pregnancies to promote fetal lung maturation^13^, we hypothesize that depleted maternal corticosteroid may be associated with offspring infection incidence through gestational age at delivery, which is closely related to lung maturation^24^. Elevated corticosteroids at T3 were also associated with better offspring lung function and showed no association with asthma. Overall, our results suggest that increased corticosteroids in the third trimester could produce multiple respiratory-related benefits. However, the effects of corticosteroid supplementation in pregnancy are not innocuous^25^, so it is important to consider risk-versus-benefit in future feasibility analyses. Additionally, increased levels of corticosteroids in the first and early second trimesters were associated with increased risk of offspring asthma. This relationship may be specific to this gestational stage, as this was not observed at T2 or T3, but stresses that other affected outcomes should be considered, as corticosteroids can have many implications for lung-related health^9,13,14^. Generally, androgens were associated with higher offspring infections and poorer lung function in this study, but comparison across multiple time periods limited interpretation due to lack of consistency. Elevated androgen levels in pregnant mothers have been linked to increased risk of pregnancy complications that can undermine fetal health^12,26^. Overall, maternal androgens appear to be associated with offspring infections, but much remains to be learned. A targeted analysis capturing a broader range of androgen metabolites may help clarify these relationships.

Our study features several strengths. Both VDAART and COPSAC collected comprehensive information on respiratory infections, asthma, and lung function metrics during follow-up, enabling comparison of multiple, related childhood outcomes^27^. The children included in these cohorts experienced a number of infections consistent with WHO observations for infants^28^, suggesting our results may be generalizable to other populations. However, VDAART and COPSAC suffered from low response to questionnaire data regarding maternal use of inhaled and oral corticosteroid medication during pregnancy, so this variable could not be explicitly included in our analyses. Maternal asthma status served as a proxy to mitigate potential confounding, and restricted analysis of non-asthmatic mothers generally reflected the same trends. Additionally, VDAART infection data was collected through non-verified caregiver reporting. While the total number of all infection types was similar per year between VDAART and COPSAC, incidence of infection subtypes (e.g., LRI, URI) differed greatly across racial and ethnic divides in VDAART. This limited our ability to investigate the role of maternal steroids in specific infection types that could be highly relevant to long-term respiratory health. Finally, follow-up studies using targeted, quantified measurement are necessary to validate the results from this study which were assessed using global, untargeted metabolomic data.

## Conclusions

Overall, this is the first study to characterize associations between maternal androgen and corticosteroid metabolites with offspring infection proneness, which represents an urgent public health issue. Clinical intervention could combat the burden of respiratory infection proneness in early life and reduce the high number of preventable deaths each year attributed to respiratory infections. Overall, our findings raise the possibility that corticosteroid supplementation in the third trimester of pregnancy may reduce offspring infection incidence with potential benefits for lung function. Future studies will address the feasibility of prenatal corticosteroid administration to reduce childhood respiratory infection proneness.

## Supplementary Information

Supplementary Information.

## Data Availability

The datasets used and/or analysed during the current study are available from the corresponding author on reasonable request.

## Abbreviations

BMI: Body mass index
COPSAC: Copenhagen prospective studies on asthma in childhood
EDTA: Ethylenediaminetetraacetic acid
FDR: False discovery rate
FEV_1_: Forced expiratory volume in 1 s
FVC: Forced vital capacity
GA: Gestational age
GW: Gestational weeks
IRR: Incidence rate ratio
IU: International units
OR: Odds ratio
RPM: Revolutions per minute
SD: Standard deviation
UPLC-MS/MS: Ultrahigh-performance liquid chromatography-tandem mass spectrometry
VDAART: Vitamin D antenatal asthma reduction trial
WHO: World Health Organization
