# Supplementary material for: Elevated third trimester corticosteroid levels are associated with fewer offspring infections
Source: Sci Rep. 2023 Jun 28;13:10461. doi: 10.1038/s41598-023-36535-0 (PMC10307773; doi:10.1038/s41598-023-36535-0)
Supplement: Supplementary file 1 — Supplementary Information. [file 41598_2023_36535_MOESM1_ESM.docx]

**Supplementary Methods**

*Metabolomic profiling in the Vitamin D Antenatal Asthma Reduction Trial (VDAART) and the Copenhagen Prospective Studies on Asthma in Childhood (COPSAC)*

Metabolon ultrahigh-performance liquid chromatography (UPLC) – tandem mass spectrometry (MS/MS) platform method^1^

Sample Preparation: Samples for metabolomic profiling were prepared using the automated MicroLab STAR® system from Hamilton Company. Recovery standards were added prior to the first step in the extraction process for QC purposes. To remove protein, dissociate small molecules bound to protein or trapped in the precipitated protein matrix, and recover chemically diverse metabolites, proteins were precipitated with methanol under vigorous shaking for 2 min (Glen Mills GenoGrinder 2000) followed by centrifugation. The resulting extract was divided into five fractions: two for analysis by two separate reverse phase (RP)/UPLC-MS/MS methods with positive ion mode electrospray ionization (ESI), one for analysis by RP/UPLC-MS/MS with negative ion mode ESI, one for analysis by HILIC/UPLC-MS/MS with negative ion mode ESI, and one sample was reserved for backup. Samples were placed briefly on a TurboVap® (Zymark) to remove organic solvent. The sample extracts were stored overnight under nitrogen prior to analysis.

QA/QC: Controls were analyzed in concert with the experimental samples: a well-characterized, pooled matrix sample generated from a representative, large number of individuals served as a technical replicate throughout the data set; extracted water samples served as process blanks; and a cocktail of QC standards that were carefully chosen not to interfere with the measurement of endogenous compounds were spiked into every analyzed sample to allow instrument performance monitoring and aid in chromatographic alignment. Instrument variability was determined by calculating the median relative standard deviation (RSD) for the standards that were added to each sample prior to injection into the mass spectrometers. Overall process variability was determined by calculating the median RSD for all endogenous metabolites (i.e., non-instrument standards) present in 100% of the pooled matrix samples. Experimental samples were randomized across the platform run with QC samples spaced evenly among the injections.

Ultrahigh Performance Liquid Chromatography – Tandem Mass Spectrometry (UPLC-MS/MS): All methods utilized a Waters ACQUITY ultra-performance liquid chromatography (UPLC) and a Thermo Scientific Q-Exactive high resolution/accurate mass spectrometer interfaced with a heated electrospray ionization (HESI-II) source and Orbitrap mass analyzer operated at 35,000 mass resolution. The sample extract was dried then reconstituted in solvents compatible to each of the four methods. Each reconstitution solvent contained a series of standards at fixed concentrations to ensure injection and chromatographic consistency. One aliquot was analyzed using acidic positive ion conditions, chromatographically optimized for more hydrophilic compounds. In this method, the extract was gradient eluted from a C18 column (Waters UPLC BEH C18-2.1x100 mm, 1.7 µm) using water and methanol, containing 0.05% perfluoropentanoic acid (PFPA) and 0.1% formic acid (FA). Another aliquot was also analyzed using acidic positive ion conditions; however, it was chromatographically optimized for more hydrophobic compounds. In this method, the extract was gradient eluted from the same aforementioned C18 column using methanol, acetonitrile, water, 0.05% PFPA and 0.01% FA and was operated at an overall higher organic content. Another aliquot was analyzed using basic negative ion optimized conditions using a separate dedicated C18 column. The basic extracts were gradient eluted from the column using methanol and water, however with 6.5 mM Ammonium Bicarbonate at pH 8. The fourth aliquot was analyzed via negative ionization following elution from a HILIC column (Waters UPLC BEH Amide 2.1x150 mm, 1.7 µm) using a gradient consisting of water and acetonitrile with 10mM Ammonium Formate, pH 10.8. The MS analysis alternated between MS and data-dependent MSn scans using dynamic exclusion. The scan range varied slighted between methods but covered 70-1000 m/z. Raw data files were archived and extracted as described below.

Data Extraction and Compound Identification: Raw data was extracted, peak-identified and QC processed using Metabolon’s hardware and software. These systems are built on a web-service platform utilizing Microsoft’s .NET technologies, which run on high-performance application servers and fiber-channel storage arrays in clusters to provide active failover and load-balancing. Compounds were identified by comparison to library entries of purified standards or recurrent unknown entities. Metabolon maintains a library based on authenticated standards that contains the retention time/index (RI), mass to charge ratio (m/z), and chromatographic data (including MS/MS spectral data) on all molecules present in the library. Furthermore, biochemical identifications are based on three criteria: retention index within a narrow RI window of the proposed identification, accurate mass match to the library +/- 10 ppm, and the MS/MS forward and reverse scores between the experimental data and authentic standards. The MS/MS scores are based on a comparison of the ions present in the experimental spectrum to the ions present in the library spectrum. While there may be similarities between these molecules based on one of these factors, the use of all three data points can be utilized to distinguish and differentiate biochemicals. More than 4,500 commercially available purified standard compounds have been acquired and registered into LIMS for analysis on all platforms for determination of their analytical characteristics.

Curation: A variety of curation procedures were carried out to ensure that a high quality data set was made available for statistical analysis and data interpretation. The QC and curation processes were designed to ensure accurate and consistent identification of true chemical entities, and to remove those representing system artifacts, mis-assignments, and background noise. Metabolon data analysts use proprietary visualization and interpretation software to confirm the consistency of peak identification among the various samples. Library matches for each compound were checked for each sample and corrected if necessary.

Metabolite Quantification and Data Normalization: Peaks were quantified using area-under-the-curve. A data normalization step was performed to correct variation resulting from instrument inter-day tuning differences by registering the medians to equal one and normalizing each data point proportionately. For this study, two distinct sample sets were merged that contained disparity in the proportions of control group composition, precluding run day normalization. Accordingly, datasets were merged by setting the medians of the control groups in the two datasets to be equal. Because sampling was kept consistent across both sample sets, this mitigates concerns that this approach artificially decreases the variance of this control group by forcing the medians to be equal.

Data processing pipeline: We calculated missingness across each metabolite and each sample, according to previously utilized methods^2^. Metabolites missing ≥ 75% were excluded. Missing values were imputed as half the minimum value across all samples for each metabolite. We examined the resulting plots of principal component analysis (PCA). Interquartile range (IQR) and skewness of each metabolite were also computed. All metabolites were subsequently log-10 transformed and pareto-scaled, and IQR and skewness were re-calculated after transformation. PCA was performed again, and distribution of PCs according to demographic variables were examined.

*Lung function measures via spirometry in VDAART children at age 6 years*

Spirometry was performed at the 6-year follow-up visits, as originally described by Litonjua.^3^ Spirometry tests were performed with the MasterScreen IOS and MasterScreen PFT systems with the Jaeger pneumotach (formerly CareFusion, now Vyaire Medical). Participants were instructed that the children should refrain from using bronchodilator inhalers, syrups, or pills for at least 8 hours before the visit and glucocorticoid inhalers or nebulizers and leukotriene modifiers for 24 hours before the visit. All results were reviewed by expert reviewers from the Pulmonary Function Laboratory of the University of Wisconsin, Madison, and acceptability and repeatability standards adhered to American Thoracic Society guidelines. For spirometry, acceptability criteria were modified to allow for a minimum expiratory time of 1 second, with a 3-second expiratory time or a plateau in the volume–time curve preferred for acceptability. For analyses, we included tests with at least two acceptable maneuvers.

*Lung function measures via spirometry in COPSAC children at age 6 years*

Forced expiratory volume in the first second (FEV1) and other spirometry measures were assessed by flow-volume curves obtained by up to five technically acceptable maneuvers in accordance with international criteria for reproducibility 4 using a MasterScope system spirometer (Erich Jäeger, Würtzburg, Germany). More details about COPSAC methods can be found at http://copsac.com/home/methods/. Spirometry was conducted at scheduled visits to COPSAC centers as part of follow-up at approximately age 6 years. Bronchial reversibility to inhaled short acting ß2-agonist (bronchodilator response) was obtained with a computer-animated volume driven incentive well-known to the children.

**Supplementary Results**

***Analyses restricted to maternal non-asthmatics***

When restricted to non-asthmatic mothers, 6 androgens showed positive significant associations with number of infections at P<0.05 for T1 in VDAART (P-value range: 2.49x10^-5^ to 0.032), and all retained significance at FDR<0.05; one androgen metabolite, 5alpha-androstan-3beta,17beta-diol disulfate, produced a significant inverse association with infection proneness (P-value: 0.032) but did not retain significance after an FDR threshold was applied. Both corticosteroids were significantly positively associated with childhood infections are a nominal significance threshold but neither survived correction for multiple testing.

In COPSAC T2, more significant associations were observed in the restricted analysis than in the overall analysis, with 8 androgenic steroid metabolites significantly positively associated with infections at P<0.05 (P-value range: 5.22x10^-5^ to 0.044; all 8 associations retained significance at FDR<0.05) and three additional androgenic steroid metabolites showed borderline significant associations at a P<0.1 threshold (P-values: 0.050 to 0.065); all significant positively associated androgen metabolites observed overall in COPSAC retained significance or became more significant in the restricted analysis. One androgenic steroid, 5alpha-androstan-3alpha,17alpha-diol monosulfate was inversely associated with infection proneness in the restricted analysis of COPSAC T2 (P-value: 0.011) and retained significance following correction for multiple testing. Neither corticosteroid was associated with infections in the restricted analysis at a nominal P<0.05 threshold.

A total of 5 androgen metabolites produced significant associations with infection proneness at VDAART T3 restricted to maternal non-asthmatics (P-value range: 2.98x10^-5^ to 0.047; 2 retained significance at FDR<0.05). Two androgenic steroids, 5alpha-androstan-3beta,17alpha-diol disulfate and 5alpha-androstan-3beta,17beta-diol disulfate, were negatively associated with infections at VDAART T3 but neither retained significance at FDR<0.05. Only cortisone was associated with infections at a P<0.05 threshold (P=0.001) and retained significance after FDR correction

**Supplemental Table 1.** Restricted analyses of non-asthmatic mothers. Incidence rate ratios (IRRs) and P-values for restricted analyses of maternal steroids and number of infections during childhood. Associations at P<0.05 are bolded. Associations at 0.05<P<0.1 are italicized for COPSAC.

| **Steroid Metabolite** | **VDAART T1 (N=774)**  **10-18 GW** | | | **COPSAC T2 (N=729)**  **22-26 GW** | | | **VDAART T3 (N=779)**  **32-38 GW** | | |
| --- | --- | --- | --- | --- | --- | --- | --- | --- | --- |
|  | **IRR (95% CI)** | **P-value** | **FDR** | **IRR (95% CI)** | **P-value** | **FDR** | **IRR (95% CI)** | **P-value** | **FDR** |
| 16a-hydroxy DHEA 3-sulfate | 1.02 (0.97,1.08) | 0.346 | 0.425 | 1.02 (0.96,1.07) | 0.532 | 0.570 | 1.02 (0.97,1.08) | 0.390 | 0.415 |
| androstenediol (3beta,17beta) monosulfate (1) | **1.08 (1.02,1.14)** | **0.011** | **0.029** | **1.1 (1.05,1.16)** | **1.54 x10^-4^** | **0.001** | **1.05 (1.01,1.1)** | **0.024** | **0.075** |
| androstenediol (3beta,17beta) monosulfate (2) | **1.09 (1.04,1.15)** | **0.001** | **0.004** | **1.08 (1.04,1.13)** | **3.02 x10^-5^** | **3.21 x10^-4^** | 1.02 (0.98,1.06) | 0.306 | 0.357 |
| androstenediol (3alpha, 17alpha) monosulfate (3) | 1.04 (0.99,1.09) | 0.124 | 0.199 | **1.06 (1.01,1.11)** | **0.021** | **0.038** | 1.03 (0.98,1.08) | 0.238 | 0.357 |
| androstenediol (3alpha, 17alpha) monosulfate (2) | **1.07 (1.02,1.12)** | **0.006** | **0.019** | **1.05 (1.01,1.08)** | **0.007** | **0.017** | **1.09 (1.05,1.14)** | **2.98x10^-5^** | **4.77x10^-4^** |
| androstenediol (3beta,17beta) disulfate (2) | **1.13 (1.07,1.2)** | **2.49x10^-5^** | **3.99x10^-4^** | **1.14 (1.07,1.21)** | **6.42 x10^-5^** | **3.21 x10^-4^** | 1.04 (0.99,1.1) | 0.143 | 0.287 |
| androstenediol (3beta,17beta) disulfate (1) | **1.08 (1.02,1.15)** | **0.005** | **0.019** | **1.11 (1.06,1.17)** | **5.22 x10^-5^** | **3.21 x10^-4^** | 1.04 (0.99,1.09) | 0.136 | 0.287 |
| 5alpha-androstan-3beta,17beta-diol disulfate | **0.95 (0.9,1)** | **0.032** | **0.063** | **1.09 (1.04,1.15)** | **0.001** | **0.002** | **0.95 (0.91,1)** | **0.047** | **0.126** |
| 5alpha-androstan-3beta,17alpha-diol disulfate | 1 (0.97,1.03) | 0.761 | 0.812 | *0.97 (0.94,1)* | *0.065* | *0.081* | **0.96 (0.92,0.99)** | **0.019** | **0.075** |
| 5alpha-androstan-3alpha,17alpha-diol monosulfate | 0.98 (0.94,1.02) | 0.311 | 0.414 | **0.94 (0.89,0.99)** | **0.011** | **0.023** | 0.98 (0.93,1.02) | 0.284 | 0.357 |
| epiandrosterone sulfate | 0.99 (0.94,1.04) | 0.631 | 0.721 | *1.05 (1,1.1)* | *0.060* | *0.081* | 0.98 (0.93,1.02) | 0.284 | 0.357 |
| andro steroid monosulfate C19H28O6S (1)* | 1.02 (0.98,1.07) | 0.307 | 0.414 | *1.05 (1,1.11)* | *0.050* | *0.075* | 0.97 (0.93,1.02) | 0.312 | 0.357 |
| dehydroepiandrosterone sulfate (DHEA-S) | **1.1 (1.04,1.16)** | **3.72 x10^-4^** | **0.003** | **1.06 (1,1.12)** | **0.044** | **0.073** | **1.09 (1.03,1.14)** | **0.001** | **0.005** |
| androsterone sulfate | 1 (0.95,1.05) | 0.995 | 0.995 | NA | NA | NA | 0.99 (0.95,1.04) | 0.783 | 0.783 |
| cortisone | **1.08 (1,1.17)** | **0.047** | **0.083** | 1.02 (0.94,1.1) | 0.648 | 0.648 | **0.9 (0.85,0.96)** | **0.001** | **0.005** |
| cortisol | **1.09 (1.01,1.18)** | **0.022** | **0.051** | *1.07 (0.99,1.15)* | *0.071* | *0.081* | 0.96 (0.9,1.02) | 0.184 | 0.326 |

**Supplemental Table 2.** Odds ratios (ORs) and P-values for analyses of maternal steroids and offspring asthma status. Associations at P<0.05 are bolded.

| **Steroid Metabolite** | **VDAART T1 (N=774)**  **10-18 GW** | | | **COPSAC T2 (N=729)**  **22-26 GW** | | | **VDAART T3 (N=779)**  **32-38 GW** | | |
| --- | --- | --- | --- | --- | --- | --- | --- | --- | --- |
|  | **OR (95% CI)** | **P-value** | **FDR** | **OR (95% CI)** | **P-value** | **FDR** | **OR (95% CI)** | **P-value** | **FDR** |
| 16a-hydroxy DHEA 3-sulfate | 1.02 (0.75,1.39) | 0.903 | 0.962 | 1.15 (0.72,1.83) | 0.566 | 0.927 | 1.09 (0.77,1.56) | 0.620 | 0.883 |
| androstenediol (3beta,17beta) monosulfate (1) | 1.18 (0.83,1.69) | 0.360 | 0.694 | 1.21 (0.81,1.8) | 0.347 | 0.699 | 1.03 (0.77,1.39) | 0.828 | 0.883 |
| androstenediol (3beta,17beta) monosulfate (2) | 1.07 (0.78,1.49) | 0.667 | 0.821 | 1.18 (0.85,1.65) | 0.314 | 0.699 | 1.05 (0.82,1.35) | 0.686 | 0.883 |
| androstenediol (3alpha, 17alpha) monosulfate (3) | 1.23 (0.9,1.67) | 0.196 | 0.694 | 1.3 (0.87,1.95) | 0.194 | 0.699 | 1.11 (0.82,1.5) | 0.514 | 0.883 |
| androstenediol (3alpha, 17alpha) monosulfate (2) | 1.11 (0.83,1.49) | 0.470 | 0.694 | 0.96 (0.71,1.28) | 0.760 | 0.927 | 0.99 (0.74,1.33) | 0.943 | 0.943 |
| androstenediol (3beta,17beta) disulfate (2) | 1.21 (0.83,1.75) | 0.321 | 0.694 | 0.98 (0.58,1.64) | 0.927 | 0.927 | 0.95 (0.65,1.39) | 0.796 | 0.883 |
| androstenediol (3beta,17beta) disulfate (1) | 1.07 (0.74,1.55) | 0.734 | 0.839 | 1.44 (0.92,2.26) | 0.111 | 0.699 | 1.11 (0.8,1.56) | 0.529 | 0.883 |
| 5alpha-androstan-3beta,17beta-diol disulfate | 1.2 (0.87,1.66) | 0.272 | 0.694 | 1.38 (0.92,2.07) | 0.125 | 0.699 | 1.13 (0.83,1.52) | 0.442 | 0.883 |
| 5alpha-androstan-3beta,17alpha-diol disulfate | 0.95 (0.78,1.15) | 0.596 | 0.795 | 1.03 (0.78,1.36) | 0.830 | 0.927 | 0.96 (0.76,1.2) | 0.704 | 0.883 |
| 5alpha-androstan-3alpha,17alpha-diol monosulfate | 0.91 (0.69,1.19) | 0.477 | 0.694 | 1.03 (0.67,1.61) | 0.879 | 0.927 | 0.9 (0.68,1.19) | 0.462 | 0.883 |
| epiandrosterone sulfate | 1.15 (0.84,1.56) | 0.382 | 0.694 | 1.3 (0.89,1.9) | 0.173 | 0.699 | 1.27 (0.95,1.72) | 0.110 | 0.883 |
| andro steroid monosulfate C19H28O6S (1)* | 0.99 (0.75,1.32) | 0.962 | 0.962 | 1.03 (0.66,1.63) | 0.883 | 0.927 | 0.95 (0.67,1.34) | 0.771 | 0.883 |
| dehydroepiandrosterone sulfate (DHEA-S) | 1.24 (0.89,1.73) | 0.211 | 0.694 | 1.22 (0.78,1.91) | 0.373 | 0.699 | 1.19 (0.86,1.65) | 0.293 | 0.883 |
| androsterone sulfate | 1.13 (0.84,1.52) | 0.429 | 0.694 | NA | NA | NA | 1.22 (0.91,1.63) | 0.180 | 0.883 |
| cortisone | **1.77 (1.1,2.84)** | **0.019** | **0.193** | 1.13 (0.59,2.13) | 0.716 | 0.927 | 0.83 (0.55,1.25) | 0.375 | 0.883 |
| cortisol | **1.77 (1.08,2.9)** | **0.024** | **0.193** | 1.39 (0.78,2.48) | 0.266 | 0.699 | 0.92 (0.61,1.39) | 0.684 | 0.883 |

**Supplemental Table 3.** Linear regression coefficients and P-values for analyses of maternal steroids and FEV_1_. Associations at P<0.05 are bolded.

| **Steroid Metabolite** | **VDAART T1 (N=774)**  **10-18 GW** | | | **COPSAC T2 (N=729)**  **22-26 GW** | | | **VDAART T3 (N=779)**  **32-38 GW** | | |
| --- | --- | --- | --- | --- | --- | --- | --- | --- | --- |
|  | **Beta (95% CI)** | **P-value** | **FDR** | **Beta (95% CI)** | **P-value** | **FDR** | **Beta (95% CI)** | **P-value** | **FDR** |
| 16a-hydroxy DHEA 3-sulfate | 0.01 (-0.03,0.04) | 0.780 | 0.957 | -0.01 (-0.04,0.03) | 0.692 | 0.798 | 0.02 (-0.02,0.07) | 0.323 | 0.706 |
| androstenediol (3beta,17beta) monosulfate (1) | 0 (-0.05,0.04) | 0.870 | 0.957 | **-0.04 (-0.07,-0.01)** | **0.012** | **0.088** | 0.01 (-0.03,0.04) | 0.658 | 0.877 |
| androstenediol (3beta,17beta) monosulfate (2) | 0.03 (-0.01,0.08) | 0.115 | 0.611 | **-0.04 (-0.06,-0.02)** | **0.001** | **0.010** | 0.01 (-0.02,0.05) | 0.400 | 0.706 |
| androstenediol (3alpha, 17alpha) monosulfate (3) | 0.02 (-0.02,0.06) | 0.372 | 0.957 | -0.02 (-0.05,0.01) | 0.169 | 0.480 | **0.04 (0,0.08)** | **0.045** | **0.240** |
| androstenediol (3alpha, 17alpha) monosulfate (2) | **-0.06 (-0.1,-0.03)** | **0.001** | **0.011** | -0.01 (-0.03,0.01) | 0.414 | 0.621 | -0.01 (-0.06,0.03) | 0.485 | 0.706 |
| androstenediol (3beta,17beta) disulfate (2) | 0 (-0.05,0.05) | 0.897 | 0.957 | -0.03 (-0.07,0.01) | 0.192 | 0.480 | 0.02 (-0.03,0.07) | 0.413 | 0.706 |
| androstenediol (3beta,17beta) disulfate (1) | 0.04 (0,0.09) | 0.080 | 0.611 | -0.03 (-0.06,0) | 0.062 | 0.308 | 0.03 (-0.01,0.07) | 0.186 | 0.612 |
| 5alpha-androstan-3beta,17beta-diol disulfate | 0 (-0.04,0.04) | 0.978 | 0.978 | -0.01 (-0.04,0.02) | 0.382 | 0.621 | -0.02 (-0.06,0.02) | 0.443 | 0.706 |
| 5alpha-androstan-3beta,17alpha-diol disulfate | 0.01 (-0.01,0.04) | 0.316 | 0.957 | -0.01 (-0.03,0.01) | 0.510 | 0.695 | 0.02 (-0.01,0.05) | 0.269 | 0.706 |
| 5alpha-androstan-3alpha,17alpha-diol monosulfate | 0.01 (-0.02,0.05) | 0.476 | 0.957 | 0 (-0.04,0.03) | 0.843 | 0.878 | 0 (-0.03,0.04) | 0.863 | 0.965 |
| epiandrosterone sulfate | 0 (-0.04,0.04) | 0.878 | 0.957 | -0.01 (-0.04,0.02) | 0.373 | 0.621 | -0.01 (-0.05,0.03) | 0.756 | 0.930 |
| andro steroid monosulfate C19H28O6S (1)* | 0.01 (-0.03,0.04) | 0.741 | 0.957 | -0.01 (-0.04,0.03) | 0.676 | 0.798 | 0 (-0.04,0.04) | 0.965 | 0.965 |
| dehydroepiandrosterone sulfate (DHEA-S) | 0.01 (-0.03,0.05) | 0.655 | 0.957 | -0.03 (-0.06,0) | 0.089 | 0.332 | 0.03 (-0.01,0.07) | 0.191 | 0.612 |
| androsterone sulfate | 0.01 (-0.03,0.04) | 0.757 | 0.957 | NA | NA | NA | 0 (-0.04,0.04) | 0.945 | 0.965 |
| cortisone | 0.02 (-0.04,0.08) | 0.548 | 0.957 | 0 (-0.05,0.04) | 0.878 | 0.878 | **0.05 (0,0.1)** | **0.036** | **0.240** |
| cortisol | 0.01 (-0.05,0.08) | 0.659 | 0.957 | -0.02 (-0.06,0.02) | 0.287 | 0.615 | **0.06 (0.01,0.1)** | **0.020** | **0.240** |

**Supplemental Table 4.** Steroid metabolites and FVC. Linear regression coefficients and P-values for analyses of maternal steroids and lung function outcomes FVC. Associations at P<0.05 are bolded.

| **Steroid Metabolite** | **VDAART T1 (N=774)**  **10-18 GW** | | | **COPSAC T2 (N=729)**  **22-26 GW** | | | **VDAART T3 (N=779)**  **32-38 GW** | | |
| --- | --- | --- | --- | --- | --- | --- | --- | --- | --- |
|  | **Beta (95% CI)** | **P-value** | **FDR** | **Beta (95% CI)** | **P-value** | **FDR** | **Beta (95% CI)** | **P-value** | **FDR** |
| 16a-hydroxy DHEA 3-sulfate | 0.01 (-0.03,0.05) | 0.693 | 0.983 | 0 (-0.04,0.03) | 0.828 | 0.955 | 0.02 (-0.03,0.06) | 0.525 | 0.835 |
| androstenediol (3beta,17beta) monosulfate (1) | 0.01 (-0.04,0.06) | 0.727 | 0.983 | -0.05 (-0.08,-0.01) | 0.008 | 0.058 | 0.02 (-0.02,0.06) | 0.317 | 0.635 |
| androstenediol (3beta,17beta) monosulfate (2) | 0.04 (0,0.09) | 0.058 | 0.466 | **-0.05 (-0.07,-0.02)** | **3.76x10^-4^** | **0.006** | 0.01 (-0.03,0.04) | 0.666 | 0.835 |
| androstenediol (3alpha, 17alpha) monosulfate (3) | 0.02 (-0.03,0.06) | 0.488 | 0.983 | -0.03 (-0.06,0) | 0.092 | 0.248 | 0.03 (-0.01,0.07) | 0.178 | 0.537 |
| androstenediol (3alpha, 17alpha) monosulfate (2) | **-0.05 (-0.09,-0.01)** | **0.017** | **0.264** | -0.02 (-0.04,0) | 0.091 | 0.248 | -0.01 (-0.05,0.04) | 0.710 | 0.835 |
| androstenediol (3beta,17beta) disulfate (2) | 0.01 (-0.04,0.07) | 0.691 | 0.983 | -0.03 (-0.07,0.02) | 0.206 | 0.387 | 0.02 (-0.04,0.07) | 0.517 | 0.835 |
| androstenediol (3beta,17beta) disulfate (1) | 0.05 (-0.01,0.1) | 0.092 | 0.489 | -0.04 (-0.07,0) | 0.056 | 0.248 | 0.04 (-0.01,0.08) | 0.149 | 0.537 |
| 5alpha-androstan-3beta,17beta-diol disulfate | -0.02 (-0.06,0.03) | 0.491 | 0.983 | -0.03 (-0.06,0.01) | 0.116 | 0.248 | -0.03 (-0.07,0.01) | 0.178 | 0.537 |
| 5alpha-androstan-3beta,17alpha-diol disulfate | 0.01 (-0.02,0.03) | 0.506 | 0.983 | 0 (-0.03,0.02) | 0.697 | 0.872 | 0.02 (-0.01,0.05) | 0.237 | 0.543 |
| 5alpha-androstan-3alpha,17alpha-diol monosulfate | 0 (-0.03,0.04) | 0.799 | 0.983 | -0.01 (-0.05,0.02) | 0.441 | 0.661 | 0 (-0.04,0.04) | 0.900 | 0.900 |
| epiandrosterone sulfate | 0 (-0.05,0.04) | 0.876 | 0.992 | -0.02 (-0.05,0.01) | 0.243 | 0.405 | -0.01 (-0.05,0.03) | 0.719 | 0.835 |
| andro steroid monosulfate C19H28O6S (1)* | 0.01 (-0.03,0.05) | 0.667 | 0.983 | 0 (-0.04,0.04) | 0.975 | 0.975 | 0.01 (-0.04,0.05) | 0.782 | 0.835 |
| dehydroepiandrosterone sulfate (DHEA-S) | 0.03 (-0.02,0.07) | 0.215 | 0.859 | -0.03 (-0.07,0.01) | 0.113 | 0.248 | 0.04 (0,0.08) | 0.074 | 0.537 |
| androsterone sulfate | 0 (-0.04,0.04) | 0.992 | 0.992 | NA | NA | NA | -0.01 (-0.05,0.04) | 0.769 | 0.835 |
| cortisone | 0.01 (-0.05,0.07) | 0.763 | 0.983 | 0 (-0.05,0.05) | 0.904 | 0.969 | 0.04 (-0.01,0.09) | 0.128 | 0.537 |
| cortisol | 0 (-0.07,0.07) | 0.934 | 0.992 | -0.01 (-0.06,0.03) | 0.588 | 0.802 | 0.03 (-0.02,0.09) | 0.201 | 0.537 |

**Supplemental Table 5.** Steroid metabolites and FEV_1_/FVC ratio. Linear regression coefficients and P-values for analyses of maternal steroids and FEV_1_/FVC. Associations at P<0.05 are bolded.

| **Steroid Metabolite** | **VDAART T1 (N=774)**  **10-18 GW** | | | **COPSAC T2 (N=729)**  **22-26 GW** | | | **VDAART T3 (N=779)**  **32-38 GW** | | |
| --- | --- | --- | --- | --- | --- | --- | --- | --- | --- |
|  | **Beta (95% CI)** | **P-value** | **FDR** | **Beta (95% CI)** | **P-value** | **FDR** | **Beta (95% CI)** | **P-value** | **FDR** |
| 16a-hydroxy DHEA 3-sulfate | -0.28 (-1.78,1.21) | 0.710 | 0.854 | 0 (-0.01,0.01) | 0.731 | 0.869 | 0.64 (-1.1,2.37) | 0.474 | 0.851 |
| androstenediol (3beta,17beta) monosulfate (1) | -0.75 (-2.43,0.94) | 0.385 | 0.854 | 0 (-0.01,0.01) | 0.785 | 0.869 | -0.67 (-2.04,0.71) | 0.342 | 0.851 |
| androstenediol (3beta,17beta) monosulfate (2) | -0.27 (-1.91,1.37) | 0.748 | 0.854 | 0 (-0.01,0.01) | 0.651 | 0.869 | 0.53 (-0.7,1.76) | 0.400 | 0.851 |
| androstenediol (3alpha, 17alpha) monosulfate (3) | 0.41 (-1.11,1.94) | 0.597 | 0.854 | 0 (-0.01,0.01) | 0.432 | 0.869 | 1.04 (-0.51,2.6) | 0.190 | 0.851 |
| androstenediol (3alpha, 17alpha) monosulfate (2) | **-1.58 (-2.99,-0.17)** | **0.029** | **0.467** | 0.01 (0,0.01) | 0.149 | 0.869 | -0.36 (-1.93,1.22) | 0.656 | 0.869 |
| androstenediol (3beta,17beta) disulfate (2) | -1.05 (-3,0.89) | 0.290 | 0.854 | 0 (-0.01,0.01) | 0.976 | 0.976 | 0.37 (-1.54,2.27) | 0.706 | 0.869 |
| androstenediol (3beta,17beta) disulfate (1) | 0.36 (-1.53,2.24) | 0.711 | 0.854 | 0 (-0.01,0.01) | 0.793 | 0.869 | -0.09 (-1.8,1.62) | 0.920 | 0.976 |
| 5alpha-androstan-3beta,17beta-diol disulfate | 1.4 (-0.25,3.05) | 0.098 | 0.773 | 0.01 (0,0.02) | 0.124 | 0.869 | 0.9 (-0.66,2.45) | 0.259 | 0.851 |
| 5alpha-androstan-3beta,17alpha-diol disulfate | 0.29 (-0.64,1.23) | 0.540 | 0.854 | 0 (-0.01,0.01) | 0.755 | 0.869 | -0.02 (-1.15,1.12) | 0.976 | 0.976 |
| 5alpha-androstan-3alpha,17alpha-diol monosulfate | 0.6 (-0.7,1.89) | 0.365 | 0.854 | 0.01 (-0.01,0.02) | 0.305 | 0.869 | 0.34 (-1.02,1.69) | 0.627 | 0.869 |
| epiandrosterone sulfate | 0.13 (-1.39,1.65) | 0.866 | 0.868 | 0 (-0.01,0.01) | 0.485 | 0.869 | 0.13 (-1.4,1.65) | 0.871 | 0.976 |
| andro steroid monosulfate C19H28O6S (1)* | -0.11 (-1.45,1.22) | 0.868 | 0.868 | -0.01 (-0.02,0.01) | 0.334 | 0.869 | -0.53 (-2.19,1.13) | 0.532 | 0.851 |
| dehydroepiandrosterone sulfate (DHEA-S) | -1.2 (-2.81,0.41) | 0.145 | 0.773 | 0 (-0.01,0.01) | 0.811 | 0.869 | -0.66 (-2.25,0.93) | 0.416 | 0.851 |
| androsterone sulfate | 0.51 (-0.94,1.96) | 0.492 | 0.854 | NA | NA | NA | 0.53 (-0.96,2.02) | 0.485 | 0.851 |
| cortisone | 0.7 (-1.61,3) | 0.554 | 0.854 | -0.01 (-0.02,0.01) | 0.502 | 0.869 | 1.4 (-0.42,3.22) | 0.132 | 0.851 |
| cortisol | 1.48 (-1.06,4.03) | 0.255 | 0.854 | -0.01 (-0.02,0.01) | 0.296 | 0.869 | 2.28 (0.43,4.13) | 0.017 | 0.267 |

|  | **Cortisone** | | | **Cortisol** | | | **Androstenediol (3alpha, 17alpha) monosulfate (3)** | | | **Androstenediol (3alpha, 17alpha) monosulfate (2)** | | |
| --- | --- | --- | --- | --- | --- | --- | --- | --- | --- | --- | --- | --- |
|  | **Beta (95% CI)** | **P-value** | **FDR** | **Beta (95% CI)** | **P-value** | **FDR** | **Beta (95% CI)** | **P-value** | **FDR** | **Beta (95% CI)** | **P-value** | **FDR** |
| Maternal age | 0.28 (-0.7,1.26) | 0.578 | 0.76 | 0.15 (-0.82,1.13) | 0.76 | 0.76 | **-1.77 (-2.47,-1.06)** | **1.17 x10^-6^** | **2.34 x10^-6^** | **-2.78 (-3.43,-2.13)** | **2.67 x10^-16^** | **1.07 x10^-15^** |
| Maternal race | **1.47 (0.99,2.18)** | **0.055** | **0.055** | **2.15 (1.44,3.21)** | **1.72x10^-4^** | **3.44x10^-4^** | **0.73 (0.56,0.95)** | **0.019** | **0.025** | **0.45 (0.34,0.59)** | **7.59 x10^-9^** | **3.04 x10^-8^** |
| Maternal ethnicity | **0.44 (0.26,0.72)** | **0.001** | **0.004** | **0.57 (0.37,0.88)** | **0.010** | **0.014** | **0.62 (0.45,0.84)** | **0.002** | **0.004** | 1.02 (0.78,1.35) | 0.86 | 0.86 |
| Pre-pregnancy BMI | **-2.27 (-3.69,-0.85)** | **0.0018** | **0.0037** | **-2.34 (-3.78,-0.89)** | **0.0015** | **0.0037** | **-1.53 (-2.59,-0.46)** | **0.0052** | **0.0070** | **1.33 (0.32,2.33)** | **0.009** | **0.009** |
| Vitamin D level | -0.01 (-2.61,2.59) | 0.99 | 0.99 | -0.28 (-2.86,2.31) | 0.83 | 0.99 | 0.13 (-1.77,2.03) | 0.89 | 0.99 | **-2.23 (-4.03,-0.43)** | **0.015** | **0.061** |
| Maternal education | 0.01 (-0.12,0.14) | 0.88 | 0.89 | 0.06 (-0.07,0.19) | 0.35 | 0.47 | -0.08 (-0.18,0.01) | 0.088 | 0.176 | **-0.3 (-0.38,-0.21)** | **9.10 x10^-11^** | **3.64 x10^-10^** |
| Birth order | **-0.28 (-0.47,-0.1)** | **0.0030** | **0.0061** | -0.04 (-0.23,0.15) | 0.67 | 0.69 | **-0.22 (-0.35,-0.08)** | **0.0019** | **0.0061** | -0.04 (-0.17,0.09) | 0.54 | 0.69 |
| Smoking in pregnancy | 0.66 (0.43,1.01) | 0.056 | 0.104 | 0.66 (0.42,1.05) | 0.078 | 0.104 | 1.08 (0.75,1.55) | 0.68 | 0.68 | **2.04 (1.36,3.08)** | **0.001** | **0.003** |

**Supplemental Table 6.** Associations between covariates included in models and metabolites significantly associated with infections at T3. Associations at P<0.05 are bolded.

**References**

1. Evans AM, DeHaven CD, Barrett T, Mitchell M, Milgram E. Integrated, nontargeted ultrahigh performance liquid chromatography/electrospray ionization tandem mass spectrometry platform for the identification and relative quantification of the small-molecule complement of biological systems. *Anal Chem*. Aug 15 2009;81(16):6656-67. doi:10.1021/ac901536h

2. Huang M, Kelly RS, Chu SH, et al. Maternal Metabolome in Pregnancy and Childhood Asthma or Recurrent Wheeze in the Vitamin D Antenatal Asthma Reduction Trial. *Metabolites*. Jan 23 2021;11(2)doi:10.3390/metabo11020065

3. Litonjua AA, Carey VJ, Laranjo N, et al. Six-Year Follow-up of a Trial of Antenatal Vitamin D for Asthma Reduction. *N Engl J Med*. Feb 6 2020;382(6):525-533. doi:10.1056/NEJMoa1906137
